# Immune Checkpoints and CAR-T Cells: The Pioneers in Future Cancer Therapies?

**DOI:** 10.3390/ijms21218305

**Published:** 2020-11-05

**Authors:** Negar Hosseinkhani, Afshin Derakhshani, Omid Kooshkaki, Mahdi Abdoli Shadbad, Khalil Hajiasgharzadeh, Amir Baghbanzadeh, Hossein Safarpour, Ahad Mokhtarzadeh, Oronzo Brunetti, Simon C. Yue, Nicola Silvestris, Behzad Baradaran

**Affiliations:** 1 Immunology Research Center, Tabriz University of Medical Sciences, Tabriz 5165665811, Iran; hosseinkhanin@tbzmed.ac.ir (N.H.); derakhshania@tbzmed.ac.ir (A.D.); abdoli.med99@gmail.com (M.A.S.); hajiasgharzadeh@tbzmed.ac.ir (K.H.); amirbaghbanzadeh@gmail.com (A.B.); ahad.mokhtarzadeh@gmail.com (A.M.); 2 Student Research Committee, Tabriz University of Medical Sciences, Tabriz 5166614766, Iran; 3 Department of Immunology, Faculty of Medicine, Tabriz University of Medical Sciences, Tabriz 5166614766, Iran; 4 Medical Oncology Unit, IRCCS IstitutoTumori “Giovanni Paolo II” of Bari, 70124 Bari, Italy; dr.oronzo.brunetti@tiscali.it; 5 Student Research Committee, Birjand University of Medical Sciences, Birjand 9717853577, Iran; omidkoshki@gmail.com; 6 Cellular & Molecular Research Center, Birjand University of Medical Sciences, Birjand 9717853577, Iran; H.Safarpour@bums.ac.ir; 7 Biotech Consulting, 44 Washington St, Brookline, MA 02445, USA; scyue1@gmail.com; 8 Department of Biomedical Sciences and Human Oncology, University of Bari “Aldo Moro”, 70124 Bari, Italy

**Keywords:** cancer therapy, immune checkpoints, immunotherapy, CAR-T cells

## Abstract

Although the ever-increasing number of cancer patients pose substantial challenges worldwide, finding a treatment with the highest response rate and the lowest number of side effects is still undergoing research. Compared to chemotherapy, the relatively low side effects of cancer immunotherapy have provided ample opportunity for immunotherapy to become a promising approach for patients with malignancy. However, the clinical translation of immune-based therapies requires robust anti-tumoral immune responses. Immune checkpoints have substantial roles in the induction of an immunosuppressive tumor microenvironment and tolerance against tumor antigens. Identifying and targeting these inhibitory axes, which can be established between tumor cells and tumor-infiltrating lymphocytes, can facilitate the development of anti-tumoral immune responses. Bispecific T-cell engagers, which can attract lymphocytes to the tumor microenvironment, have also paved the road for immunological-based tumor elimination. The development of CAR-T cells and their gene editing have brought ample opportunity to recognize tumor antigens, independent from immune checkpoints and the major histocompatibility complex (MHC). Indeed, there have been remarkable advances in developing various CAR-T cells to target tumoral cells. Knockout of immune checkpoints via gene editing in CAR-T cells might be designated for a breakthrough for patients with malignancy. In the midst of this fast progress in cancer immunotherapies, there is a need to provide up-to-date information regarding immune checkpoints, bispecific T-cell engagers, and CAR-T cells. Therefore, this review aims to provide recent findings of immune checkpoints, bispecific T-cell engagers, and CAR-T cells in cancer immunotherapy and discuss the pertained clinical trials.

## 1. Introduction

Cancer is the second-largest reason for mortality after cardiac disease, with a world incidence and mortality of about 14.1 million and 8.2 million deaths per year, respectively [1]. This disease is characterized by excessive proliferative signaling, cell death resistance, evasion of growth suppressors, angiogenesis activation, invasion activity, and metastasis, and it can block the function of some genes to avoid the immune system and form a tumor [2,3,4]. In cancer, disrupted cell pathways and tumor-specific DNA modifications contribute to the development of new neoantigens, which can be identified by immune cells, especially T cells [5]. Cytotoxic T cells (CTLs) have a pivotal role in controlling and removing cancerous cells [6].

**Figure 1:**
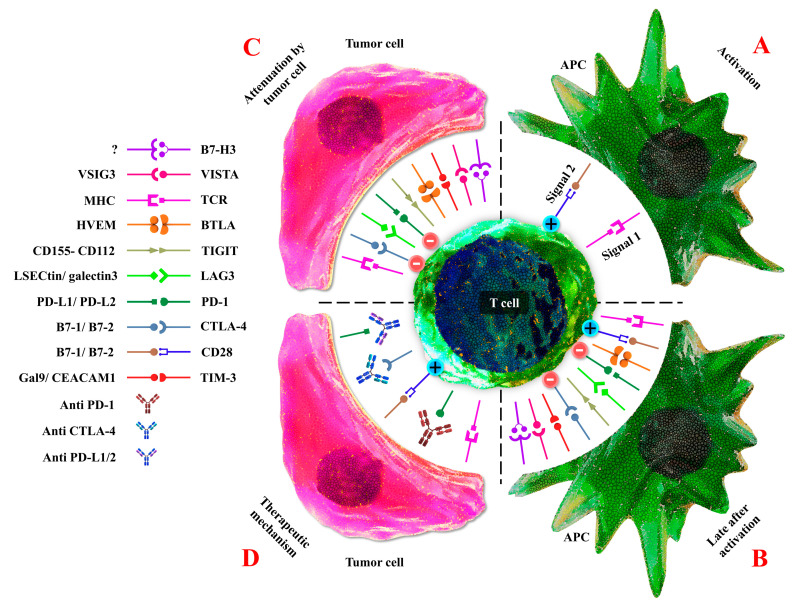
Mechanisms of ICIs. (**A**) Activation of T cells following two signals, the first interaction between TCR and MHC and the second, co-stimulatory signals (CD28 and B7-1/B7-2). (**B**) Expression of ICIs and interaction with their respective ligands to maintain immune homeostasis. (**C**) Cancer cells express increased inhibitory ligands to bind coinhibitory receptors on T-cells that lead to immune suppression. (**D**) mAbs that block ICIs such as CTLA-4, PD-1, PD-L1 restore T-cells effector function. (Abbreviations: ICI: Immune checkpoint inhibitors, TCR: T-cell receptor, MHC: Major histocompatibility complex, mAb: Monoclonal antibody, CTLA-4: Cytotoxic T-lymphocyte-associated protein 4, PD-1: Programmed cell death protein 1, PD-L1: Programmed death-ligand 1).

There is a wide variety of intricate connections between cancer cells, immune cells such as T cells, antigen-presenting cells (APCs), B cells, natural killer (NK) cells, and tumor stroma. Activation of T lymphocytes and associated effector activity development are based on at least two signals from APCs [7]. The first is generated by a peptide major histocompatibility complex (MHC) and T-cell receptor (TCR) interactions. The second is a costimulatory signal mediated by the engagement of T cell surface molecules with their ligands that are expressed on APCs, such as the interplay between CD28 on T cells and either B7-1 (CD80) or B7-2 (CD86) on APCs [8] (Figure 1A). The cancer-related immune response is a consequence of interaction within stimulating and inhibitory signals. Immune checkpoints (ICs) are critical regulators of immune systems that preserve immune homeostasis providing self-tolerance through the control of the type, intensity, and period of the immune response. In physiological conditions, ICs enable the immune system to respond to host antigens preserving healthy tissues. On the other hand, these molecules are responsible for tumor cell evasion in different types of cancers. These proteins, as negative modulators, express on tumors and promote the extension of cancer cells [2,9]. In general, cytotoxic T lymphocyte antigen-4 (CTLA-4) and programmed cell death protein 1 (PD-1) are two essential ICs that were previously identified as molecules performing a function in apoptosis, T cell activation, and the preservation of acquired immune system tolerance (Figure 1B). There are many challenges in the use of these molecules. Many different monoclonal antibodies that can block immune checkpoints have appeared as potent agents in the oncological models. Several studies confirmed that inhibition of ICs by immune checkpoint inhibitors (ICIs) and their application as single agents or as supplementary therapy are effective treatments in cancers [10,11]. Chimeric antigen receptor T (CAR-T) cells are a class of immunotherapy that works by employing altered T cells to fight against cancer. CAR T-cell treatment requires a genetic alteration of the autologous T-cells of the patients to produce a tumor antigen-specific CAR following ex vivo extension and then returned to patients via infusion [12]. Therefore, immunotherapy, which triggers the immune system to indirect tumor killing, has become a promising antitumor strategy, after surgical oncology, radiotherapy, chemotherapy, and target therapies. In particular, ICIs and CAR-T cells are recently approved emerging therapies in treating several cancers [13]. Monoclonal antibodies (mAbs) improve the immune response of CTLs by blocking the ICs on T cells or their ligands on APCs and cancer cells [14,15]. Bispecific T-cell engagers (BiTEs) are a novel generation of immunotherapy for cancer treatment. BiTE, as a recombinant bispecific antibody, comprises two related single-chain variable fragments (scFvs) from two separate antibodies, one of them against the T cell’s surface proteins and the other against the cancer cells antigens [16]. In preclinical studies, combination therapy of ICIs and CAR-T cells has improved efficacy compared to each treatment alone in several malignancies; therefore, their usage in clinical studies can be promising [17]. This review mainly discusses the mechanisms of the ICIs and CART cells and the combination of them in cancer therapy. Moreover, we mentioned BiTE molecules as a new immunotherapeutic molecule in cancer treatment.

## 2. Immune Checkpoints Inhibitors: The Pioneering Immunotherapy

**Figure 2:**
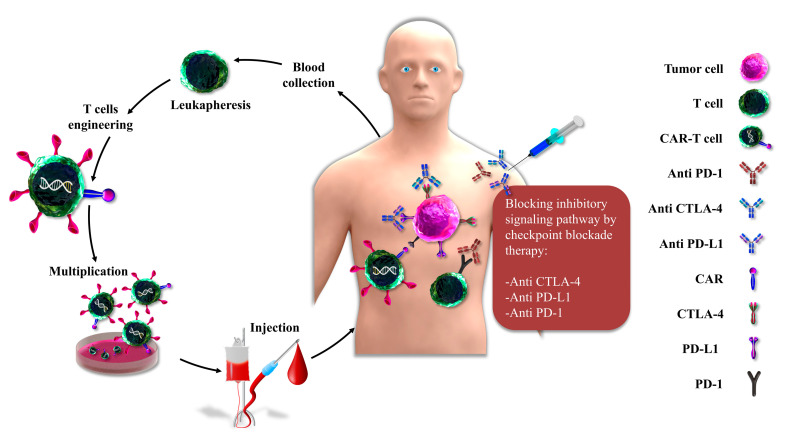
Combination of ICI blockade and CAR-T cell therapy. Collecting blood and separating leukocytes by leukapheresis, genetic manipulation of T cells, multiplication of produced engineered CAR-T cells, injection of CAR-T cells, and blocking the inhibitory signaling pathways by mAbs such as anti-CTLA-4, anti-PD-1, and anti-PD-L1 for enhancing therapeutic effects. (Abbreviations: ICI: immune checkpoint inhibitors, CAR-T: Chimeric antigen receptor T, CTLA-4: Cytotoxic T-lymphocyte-associated protein 4, PD-1: Programmed cell death protein 1, PD-L1: Programmed death-ligand 1).

ICs are cell-surface proteins located primarily, but not specifically, on T cells and NK cells. They can have a negative or positive function in the lymphocyte recruitment after the identification of appropriate ligands on the APCs or target cells. ICs, through interaction between ligands and their related receptors on the effector and target cells, can enhance stimulatory or inhibitory signals. ICs are important factors in regulating immune homeostasis and the prevention of autoimmunity [18]. Moreover, some cancer cells display an increased inhibitory ligand expression able to bind co-inhibitory receptor molecules leading to immune-response suppression. So, they result in a decrease in responses to the tumor cells and an increase in cancer cell evasion from immune cells [19,20]. The inhibition of these ICs restores the immune response against cancer cells [9]. ICIs lead to immune system reactivation, tumor shrinkage, and decreased dissemination by breaking the tolerogenic immune environment [7,21]. Conversely, these antibodies restore the equilibrium toward anti-cancer innate and adaptive response [20,22]. Furthermore, mAbs targeting co-inhibitory ICs (i.e., PD-1 and CTLA-4) have shown clinical efficacy in several cancers, such as melanoma [23], non-small cell lung cancer (NSCLC) [24], renal and bladder cancers [14], head and neck cancer [25], colorectal carcinoma (CRC) [26,27] hepatocellular carcinoma (HCC) [28], Merkel cell carcinoma [29], and Hodgkin lymphoma [30]. ICIs represent the new state of the therapy for a wide spectrum of FDA-approved indications Table 1. Other ICs include lymphocyte activation gene-3 (LAG-3), T cell immunoglobulin and mucin-domain containing-3 (TIM-3), T cell immunoglobulin and immunoreceptor tyrosine-based activation motif (ITIM) domain (TIGIT), V-domain Ig suppressor of T cell activation (VISTA), B7 homolog 3 protein (B7-H3) and B and T cell lymphocyte attenuator (BTLA), with the possibility of clinical applications as promising therapeutic targets [31] (Figure 2).

### 2.1. Mechanisms of CTLA-4 Blockade for Cancer Treatment

CTLA-4 that belongs to the CD28 family receptors is a homolog of CD28 with oppositional functions. T-cells can express both the mentioned receptors and bind to B7-1 and B7-2 ligands on APCs (Table 2). While CD28 interacts with B7-1 and B7-2, intracellular signals via phosphatidylinositol-3kinase (PI3-K) lead to enhancement of T-cells proliferation, differentiation, and survival [20,33]. CTLA-4 and CD28 compete for binding to B7-1 and B7-2 on APCs such as dendritic cells, B lymphocytes, and other immune cells [34]. The association of CTLA-4 with Src homology 2-containing phosphotyrosine phosphatase (SHP2) causes CD3ζ chain dephosphorylation, limiting the TCR′s signaling potential and improving peripheral tolerance [35]. CTLA-4 recruitment of Protein Phosphatase 2A (PP2A) leads to a decrease in Akt phosphorylation that leads to a decline in T cell activation, which is triggered by the interaction of TCR by antigens [36]. CTLA-4 functional inhibition acts in a signal independent manner. Through trans-endocytosis, CD80 and CD86 will be removed from the APC surface by CTLA-4 (5). Regulatory T-cell (Treg) mediates the function of CTLA-4 [37,38]. Lack of CTLA4 in Tregs is the cause of T-cell activation and autoimmunity [39,40]. CTLA-4 clinical trials led to FDA approval of ipilimumab and prompted further investigations on melanoma metastasis. After a second CTLA-4 mAb, tremelimumab was developed [41]. Ipilimumab and tremelimumab are CTLA-4-targeting monoclonal IgG antibodies, IgG1, and IgG2 antibody subclasses, respectively. Although ipilimumab has a higher rate of dissociation, both ipilimumab and tremelimumab have identical binding affinity to CTLA-4. Epitopes between mAbs are similar because both of them bind to CTAL-4 molecule F and G strands [42]. The inhibitory effect of each mAbs on CTLA-4 promotes the binding of CD28/B7 and enhances T cell proliferation and immune response [43,44]. Another suggested mechanism by CTLA-4 mAbs is the Treg exhaustion in the tumor microenvironment (TME) [45,46]. Nevertheless, conflicting results have been reported by other studies, not confirming a Treg depletion in CTLA-4 mechanisms of action [47,48].

### 2.2. Mechanisms of PD-1/PD-L1 Blockade for Cancer Therapy

Honjo et al. first identified PD-1 which belongs to the immunoglobulin gene superfamily [51]. Subsequently, PD-1 was recognized to be a receptor triggering-cell death in activated T-cell hybridoma [76]. PD-1 is a transmembrane protein, expressed in special subsets of thymocytes and T cells, particularly after stimulation of antigen receptors [27]. Moreover, PD-1 is also expressed on non-T cell subsets, such as B cells, APCs, and NK cells [51]. Leading to an intricate transformation of epigenetics and transcription in T cells, and forming a unique, hyporesponsive phenotype named “exhaustion” [49]. T cell exhaustion is a phenomenon of a loss of function that results from prolonged antigen stimulation in a chronic environment such as TME. Exhausted T cells are also distinguished by PD-1, TIM3, and LAG3 co-expression [77]. An important difference is that, although T cells remain in an inactive form, their harbor capacity is decreased [78]. PD-1 has two ligands, PD-ligand-1 (PD-L1) (identified as CD274 or B7-H1), generally expressed by many somatic cells primarily when exposed to pro-inflammatory cytokines, and PD-L2 (also identified as B7-DC or CD273), with more limited antigen-presenting expression [52]. Furthermore, PD-L1 cooperates with the CTLA-4, and CD80, to restrain T cell proliferation [79]. PD-1 is functionally important for homeostatic peripheral tolerance maintenance, as demonstrated by the autoimmune pathologies arising from genetic deletion of Pdcd1 (PD-1 encoding gene). For instance, a genetic lack of Pdcd1 causes lupus-like autoimmune pathology and autoimmune dilated cardiomyopathy in C57BL/6 and BALB/c mice, respectively [78]. This signaling pathway activation decreases inflammatory cytokines and protein production that influence cell survival [36,80]. Surprisingly, recent studies demonstrate that SHP2 is not necessary for anti-PD-1 or T-cell exhaustion responses in vivo [81]. Most cancer cells upregulate surface expression of PD-L1 to escape immune surveillance [82]. Antibodies targeting PD-L1 or PD-1 can block T cell anergy and re-sensitize cancer cells to anti-tumor immunity [83,84]. FDA has currently approved some monoclonal antibodies target the PD-L/PD-L1 axis (atezolizumab, durvalumab, nivolumab, cemiplimab, and pembrolizumab) to manage several cancers [50]. Some clinical trials are assessing the efficiency of the combination anti-PD-1/PD-L1 and anti-CTLA-4 for instance, A phase I/II study evaluating the efficacy and safety of AK104, a bispecific antibody against CTLA-4 and PD-1 in relapse/refractory T cell lymphoma, melanoma, and advanced solid tumors (NCT04444141; NCT04172454). For treating NSCLC, a phase I/II clinical trial is determining the efficiency of Nivolumab and Ipilimumab in combination with chemotherapy (NCT04043195).

## 3. Beyond CTLA-4 and PD-1/PD-L1 Inhibition: New Immune-Landscape to Break Cancer

### 3.1. LAG-3

Triebel et al. discovered LAG-3 (CD223) in 1990 [85]. The LAG-3 gene is close to the CD4 gene and further analysis revealed that about 20% of its amino acid sequences are identical to CD4 [31,86]. Just like CD4, LAG3 binds with a much higher affinity to MHC class II; therefore, these results indicate that LAG3 may also have been developed from a gene duplication of the CD4 locus [86]. Several possible ligands have appeared over the past five years (Table 1), A 31 kDa galactose-binding lectin named Galectin-3 (Gal-3) which regulates the activation of T cells, binds LAG3, leading to suppression of CD8+ T cells function. Gal-3 can be expressed in various types of cells using several pathways to exercise its modulatory role on CD8+ T cells [53,54]. The sinusoidal endothelial cell C-type lectin (LSECtin) is present in the liver and lymph nodes. It belongs to the DC-SIGN family molecules and is suggested as a ligand for LAG3. This molecule binds to LAG3′s four glycosylated sites. LSECtin is expressed in melanoma and liver cancer cells, suggesting a process that allows LAG3 to regulate NK cells and CD8+ T cells in TME [57]. Fibrinogen-like protein 1 (FGL1) has recently been recognized as a new LAG3 ligand [56]. LAG-3 is expressed after MHC class II binding by T cells and NK cells [22]. Although its functional mechanism is not well known, it has been recognized to have a reverse regulatory role in the activity of T cells, preserving tissues and preventing autoimmunity. After infiltrating tumor-specific lymphocytes, co-expression of LAG-3 and PD-1 causes immune exhaustion and growth of the tumor [87]. Therefore, the LAG-3 blockade increases immunity against tumor cells. Furthermore, by its different pathways of function, it is mostly regulated by preventing the cell cycle progression; it can improve the efficacy of other forms of immunotherapy [22]. While simultaneous use of anti-LAG-3 with anti-PD-1 treatment is deemed synergistic, it is unknown whether other ICIs will be as effective in combination with anti-LAG-3 therapy [88]. A phase I/II clinical study is evaluating anti-tumor efficacy and safety of anti-LAG-3 monoclonal antibody, BMS-986016 alone, or in combination with anti-PD-1 antibody (NCT01968109). Another phase I/II clinical trial is assessing the effective combination of anti-LAG-3, anti-PD-1, and anti-TIM-3 in melanoma (NCT04370704). Two inhibitory types of drugs are currently being developed. The first is mAbs targeting LAG-3 (such as BMS-986016, or Relatlimab, LAG525, or IMP701, REGN3767, and TSR-033) [55]. The use of mAbs against LAG-3 interferes with the interaction of LAG-3 between tumor and/or immune cell that express MCH II molecules and promotes tumor cell apoptosis. The latter is represented by LAG-3-Ig fusion proteins such as IMP321 or eftilagimod alpha (Immuntep^®^), a soluble form of LAG-3, induces the co-stimulative molecules and enhances the production of interleukin (IL)-12 to boost tumor immunity [31,55,60].

### 3.2. TIM-3

TIM-3, also known as hepatitis A cellular receptor 2 (HAVCR2), has some specific properties that make it another interesting IC [58]. TIM-3 was reported as a molecule specifically expressed on CD8+ T cytotoxic 1 (Tc1) and CD4+ T helper1 cells. Nowadays, it is generally known as an IC molecule [31]. It is a direct negative T-cell regulator and expressed in some other immune cells. By exciting the proliferation of myeloid-derived suppressor cells (MDSCs), TIM-3 induces immunosuppression indirectly. The rates of TIM-3 in dysfunctional and exhausted T cells are high, which indicates a significant role in malignancy [59], on the other hand, low rates of TIM-3 have been shown in autoimmune disorders such as multiple sclerosis or diabetes. Furthermore, using mAbs against TIM-3 leads to T cell proliferation and cytokine secretion, which can explain the anti-tumor role of it as well as its significance in the aggravation of autoimmune disorders [89]. TIM-3 and PD-1 associated upregulation, and TIM-3+/PD-1 + lymphocytes have the most exhausted phenotypes that cause decreased T cell proliferation and a reduction of cytokine releases such as IL-2, tumor necrosis factor (TNF), and interferon-gamma (IFN-γ) [60]. This pathway is modulated by several ligands (Table 1), such as galectin-9, phosphatidylserine, carcinoembryonic antigen-related cell adhesion molecule-1 (CEACAM-1), and high-mobility group box-1 (HMGB-1) [60,87]. These molecules are important in cancer and have a pivotal impact on different tumors’ survival and progression [22]. TIM-3, along with other inhibitory mechanisms, interrupt cellular function by modulating cell apoptosis [90]. This might reveal the synergistic effect when using with other ICIs. A dual blockade of TIM-3 and PD-1 in the murine model showed a promoted adaptive resistance compared with PD-1 monotherapy [91]. In this context, phase I clinical trials are assessing the safety and efficacy of anti-TIM-3 mAbs (i.e., Sym023; TSR-022; INCAGN02390; LY332I367; MBG453; BGBA425). Furthermore, some other trials are assessing the combination of TIM-3 with anti-PD-1 mAbs, such as anti-TIM-3/PD-L1 and anti-TIM-3/PD-1 bispecific antibodies (i.e., LY3415244 and RO7121661), for therapy to apply to solid and hematological malignancies [60,92,93]. A phase II clinical trial is determining the efficacy of TSR-022, as an anti-TIM-3 antibody in combination with TSR-042 (anti-PD-1 antibody) in eradicating tumor cells in advanced or metastatic liver cancer (NCT03680508). The effectiveness of the combination of INCMGA00012 (anti-PD-1 antibody), INCAGN02385 (anti-LAG-3 antibody), and INCAGN02390 (anti-TIM-3 antibody) is under evaluation in a phase I/II clinical study in melanoma patients (NCT04370704).

### 3.3. TIGIT

Yu and his colleagues for the first time determined TIGIT as an immune checkpoint that suppresses T cell activation in 2009 [63].

VSTM3 or TIGIT is one of the primaries for the maturing process of Treg from naive T cells [60]. This protein (also known as WUCAM) is a member of the CD28 family-like receptor and is expressed on T and NK cells (Table 2) [94]. TIGIT in combination with anti-inflammatory (e.g., IL-10) and limiting inflammatory cytokines (IFN-γ and IL-17) release an immunosuppressive function on T and NK cells and reduce dendritic cells’ (DC) maturation [63,95]. TILs frequently express high TIGIT levels in addition to PD-1, TIM-3, and LAG-3 that are a feature of the dysfunctional phenotype [61]. Furthermore, the exhausted phenotype of CD8+ TILs expresses PD-1, TIM-3, and TIGIT simultaneously. Dual blocking of PD-1/PD-L1 (and/or TIM-3) and TIGIT could be a beneficial method for restoring CD8+ TILs functionality. In the CT26 murine CRC model [96], inhibiting both TIGIT and PD-1 triggered a tumor rejection and reversed CD8+ T cell exhaustion. Therefore, this dual inhibition enhanced proliferation, cytokine secretion, and degranulation process in CD8+ TILs in melanoma subjects. According to these results, a variety of phase I trials with blocking TIGIT or combined with PD-1 or PD-L1 blocking are underway in solid tumor subjects [60,62]. For instance, phase I clinical studies are determining tumor-killing efficacy of IBI939, as an anti-TIGIT mAb alone or in combination with Sintilimab (anti-PD-1 mAb) in advanced malignancies (NCT04353830), or AB154, as an anti-TIGIT mAb in combination with Zimberelimab (anti-PD-1 mAb) in solid tumors (NCT03628677).

### 3.4. VISTA

VISTA, a type I transmembrane protein, also recognized as PD-1 homolog (PD-1H), is a specific IC with a dual function. On the one hand, VISTA triggers immune activation as a stimulating ligand for APCs and on the other hand, inhibits T cell activation, and releases cytokines as a negative ligand for T cells [65]. VSIG-3 is a new ligand for VISTA and also VSIG-3′s binding to VISTA in activated T cells prevents the proliferation of T-cells and the production of cytokines and chemokines [67]. VISTA does not have a normal cytoplasmic ITIM or immunoreceptor tyrosine-based switch motif (ITSM). Two protein kinase C binding sites and a proline-rich motif that can act as docking sites, in the intracellular tail indicate that VISTA can act as a receptor and ligand [97]. Le Mercier et al. showed that blocking VISTA could modify the suppressive characteristic of the TME by improving the infiltration, proliferation, and effector function of TILs in preclinical studies of multiple mice models [64]. In several cancers, the level of VISTA expression is different, but it has proven that the blocking of this molecule is effective even in unmeasurable levels that make it an applicable marker for clinical administrations, but finding a unique biomarker to predict the response remains a challenge [66]. Furthermore, the primary expression of this molecule by TILs makes it more tumor-specific and with less toxicity than other cascades. The anti-tumor effectiveness of CI-8993, as an anti-VISTA mAb, is being evaluated in a phase I clinical trial in solid tumors (NCT04475523). In another phase I clinical trial, two VISTA targeted molecules were investigated: JNJ-61610588, a fully human mAb anti-VISTA, and CA-170, an oral inhibitor of both PD-L1/PD-L2 and VISTA [22]. For pancreatic cancer patients, VISTA has been seen as a promising target for immunotherapeutic interventions based on the latest results revealed by Blando et al. They showed VISTA is an immune checkpoint particularly expressed in pancreatic cancer at higher rates [98].

### 3.5. B7-H3

B7-H3 or CD276, a protein of the B7-CD28 family, is commonly expressed in immune cells such as APCs, NK, B, T cells, and also various solid organs. Previously, studies reported uncovered B7-H3 as a co-stimulator because it could induce the response of T cells and the release of IFN-γ, but other investigations have reported that it leads to a negative effect on T cell activation, proliferation, and the release of cytokines [70,71]. It was overexpressed in several tumor tissues (e.g., melanoma, NSCLC, prostate, pancreatic, ovarian cancer, and CRC) and associated with disease states and prognosis [69,99]. Therefore, the B7-H3 blockade would be beneficial to improving innate immunological responses to tumor cells and impact tumor behavior directly [69,71]. Furthermore, compared to other anti-cancer strategies, a combination of the B7-H3 blockade with chemotherapy or other ICIs seems to be effective. Enoblituzumab (MGA271), a new-engineered IgG1 mAb has been identified to block B7-H3 (NCT01391143) [68,100]. Another agent is MGD009 that is a humanized dual-affinity re-targeting (DART). DART is the use of molecules which recognize both CD3 on T cells and B7-H3 on target cells. It was determined to redirect T cells to the tumor area in order to destroy targeted cells [72]. MGC018, as an -anti-B7-H3 mAb, is assessed alone or in combination with MGA012 (anti-PD-1 mAb) in a phase I/II clinical trial in advanced solid tumors patients (NCT03729596).

### 3.6. BTLA

The B and T lymphocyte attenuator (BTLA), also called CD272, with blockade properties, has similarities in structure and function with CTLA-4 and PD-1. BTLA′s structure comprises a single extracellular region, a transmembrane, and a cytoplasmic tail that contains ITIM and ITSM. Through recruiting the SHP-1 and SHP-2, and sending a negative signal to T cells, BTLA is expressed on mature lymphocytes (such as B cells, T cells, and Tregs), macrophages, and mature bone marrow-derived DC [73,74]. The BTLA ligand, a herpes virus entry mediator (HVEM), which belongs to the TNF receptor superfamily, blocks the activation, proliferation, and cytokine release of B and T cells. This pathway is manipulated by tumor cells by stimulating the production of the dysfunctional phenotype of T cells that constantly express BTLA that makes T cells inactivate. Furthermore, tumor cells as seen in melanoma express HVEM and inhibit T cells’ function by interaction with BTLA [75]. High BTLA/HVEM rates in patients with melanoma and gastric cancer are associated with poor prognosis. Therefore, the BTLA/HVEM cascade is assumed as an innovative therapeutic target for ICI blockade therapy [22]. Ongoing clinical trials are evaluating the anti-tumor activity of recombinant humanized anti-BTLA mAbs including JS004 in advanced solid tumors (NCT04278859) and recurrent/refractory malignant lymphoma (NCT04477772) and TAB004 in metastatic and unresectable solid tumors (NCT04137900).

## 4. Bispecific T-Cell Engager (BiTE) Molecules

**Figure 3:**
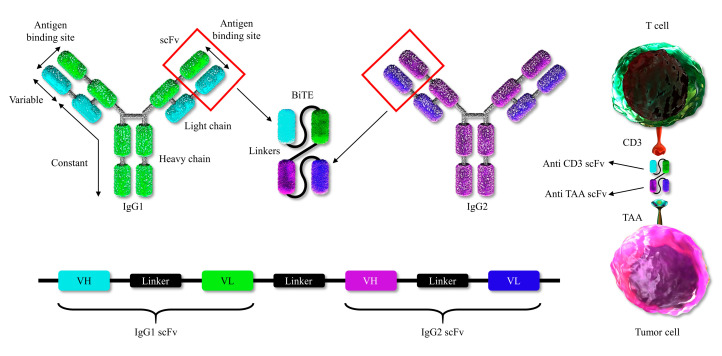
Engineering of a bispecific T cell engager (BiTE) antibody. Production of a BiTE antibody from the variable domains of two separate monoclonal antibodies (see text for details).

BiTE, a bispecific T cell engager, is an artificial bispecific antibody made up of two linked scFvs, one arm recognizing CD3 component of the receptor (such as CD3ε) on T-cells and a second one targeting tumor-associated antigen. BiTEs direct CTL to the targeted cancer cell and induce tumor cell lysis by CTL. BiTE-mediated interactions are completely independent of the MHC haplotype [101] (Figure 3).

A bispecific CD33/CD3 BiTE antibody (AMG330) has been produced to manage the relapsed/refractory acute myeloid leukemia (AML) subjects (NCT02520427). Since CD33 is over-expressed in AML blasts, a BiTE antibody was developed against both CD3 and CD33 to induce T cells to kill CD33 + AML cells, so AMG330 is very effective in preclinical studies. However, some patient samples showed minimal T-activation and reduced tumor cell lysis [102]. A major cause that limits the therapeutic efficacy of AMG330 was the presence of PD-L1 expression on AML blasts. Therefore, combining PD-L1/PD-1 inhibition with CD33/CD3 BiTE antibody causes a significant promotion in AMG 330-mediated lysis [102].

## 5. Cell Therapy with Chimeric Antigen Receptor T (CAR-T)-Based Strategies

Cell therapy with CAR-T is an innovative strategy for fighting cancer by genetically engineering patient T cells that particularly recognize and suppress targeted cells [103]. CAR-T cells identify the tumor-associated antigen (TAA) independently of the MHC [104]. The project for CAR-T cell development aims to link an extracellular ligand recognition domain, generally an scFv, to an intracellular signaling system that includes triggering T cells’ function with CD3ζ [105]. The first-generation of CARs was engineered to include just one intracellular signal region [106], with a slight anti-tumor impact and poor in vivo persistence and effectiveness due to insufficient receptor co-stimulation. In the second generation of CARs, anti-cancer activity was improved by joining CD28 or 4-1BB as a co-stimulatory domain [103], CD28 co-stimulation induces an effective but short-lived effector-like phenotype, whereas 4-1BB produces stronger expansion, longer in vivo persistence, and enhanced efficiency to produce a central memory T cell [107]. In comparison, CD28-expressing CAR-T cells have more IL-2 secretion that gives rise to faster tumor eradication [108]. This generation is the most used cancer therapy because of its stable action, manageable side effects, and a more extensive clinical experience of malignancy therapy. After constant stimulations, CD45RO+ CCR7- effector memory cells and CD45RO + CCR7 + central memory cells are formed from CD28 and 4-1BB CAR-T cells, respectively [109]. The third generation of CARs consists of activated domains and multiple co-stimulative domains; for example, joining the two domains of CD28, CD27, and OX40 (CD134), boosts the efficiency of CAR T cells to recognize tumor cells. Moreover, they improve the killing action of T-cells on tumor cells. It also showed the second and third generations of CARs increase the cytokine release as well as the proliferation of CAR-T cells in vitro. Lastly, the fourth generation of CARs has enhanced the effect of CAR-T therapy, like the addition of suicide genes and the expression of pro-inflammatory cytokines like IL-12, 15, 18 [103,105]. A phase I clinical trial evaluated a CAR-T cell against MUC16ecto in ovarian carcinoma, which has been modified to secret IL-12. However, reported therapeutic benefits were little but no adverse effects were observed [110,111]. Another pro-inflammatory cytokine is IL-18 that augment IFN-γ production [112]. IL-18 CAR-T cells against CD19 + melanoma cells showed better anti-tumor efficacy in comparison with CAR-T cells without IL-18 release [113]. Furthermore, it has been revealed that IL-18 derives CAR-T cells’ polarization into T-bet ^high^ FoxO1 ^low^ effector cells that trigger an acute inflammatory response against tumor cells [114]. The novel fifth-generation CAR constructs have been currently explored, which is similar to second-generation CARs with CD3ζ and costimulatory domain, and additionally contains a truncated cytoplasmic domain from the IL-2 receptor β chain and a STAT3/5 binding tyrosine-X-X-glutamine (YXXQ) motif. This study has shown that fifth-generation CAR-T cells have antigen-independent activities and more proliferation, persistence, and anti-tumor effects [115]. The FDA approved two second-generation CD19-specific CAR-T cell therapies for B-cell cancers in 2017 as follows:

(a) Tisagenlecleucel for adults with refractory, diffused large B-cell lymphoma (DLBCL), and refractory acute lymphoblastic leukemia (ALL) [116,117], based on phase II ELIANA trial with 82.5% remission rate [118].

(b) Axicabtagene ciloleucel for patients with DLBCL, primary mediastinal B-cell lymphoma, and high-grade B-cell lymphoma that results from follicular lymphoma [118,119], based on a multi-center clinical trial (ZUMA-1) with a 52% remission rate in refractory DLBCL subjects [120].

However, in some cases, malignant B cells lose or downregulate CD19 so, treatment with CD19 specific CAR-T cells in this type of cancers is not effective anymore [121,122]. Some other targets are currently being evaluated for B cell lymphoma, such as CD20 (NCT03576807, NCT03664635), CD22 [123,124], receptor tyrosine kinase-like orphan receptor 1 (ROR1) (NCT02706392), and Ig kappa chain [125], to address these challenges.

In cancer immunotherapy, NK cells can be applied for the production of CAR products. In comparison with T cells, CAR-NK cells do not require HLA matching and also appear to have lower allergenicity; but in just the same way, NK-mediated GVHD has been reported [126]. Nevertheless, there are some complications with the use of CAR-NK cells, such as NK cells’ low persistence and complex signaling system [127]. Invariant NKT cells (iNKT) as a subset of T cells with limitations in lipid antigens (which is presented by CD1d) in recognition by TCR, were used to manufacture CAR. Anti-CD19 CAR-iNKT against lymphoma cells with CD1d and CD19 expression have demonstrated increased anti-tumor activity [128].

## 6. Allogeneic CAR-T

Autologous CAR-T cells can be applied to prevent allogeneic responses such as rejection of CAR-T cells or Graft versus host disease (GVHD), which leads to the final, long persistence of engineered T cells [129]. However, there are some challenges to autologous CAR-T cell treatment, first of all, the cost of the process; secondly, gathering sufficient amounts of lymphocytes from patients with lymphopenia following treatment with chemotherapy or because of the underlying disease. Thirdly, manufacturing takes about 2–3 weeks, and some patients may show disease progression in this time [130]; autologous T-cells in some patients may not be effective because of T-cell dysfunction which could occur in some malignancies due to tumor characteristics or TME-derived immunosuppression mechanisms [131]. Therefore, using healthy donor-derived allogeneic CAR-T-cells may resolve these problems. The use of gene-edited allogeneic CAR-T cells can prevent GVHD as well. Furthermore, a healthy donor, gene-edited CAR-T cell that does not need matching HLA can be used to prepare an “off the shelf” product, which considers the challenges and limitations that can emerge from manufacturing CAR-T cells from T cells of the subjects [130].

### 6.1. CAR-T Cell Manufacturing from A Stem Cell Transplant Donor

Hematopoietic Stem Cell Transplant (HSCT) with an HLA-matched donor is a standard therapy for patients with B-cell ALL; therefore, because of donor and patient HLA matching, CAR-T cells received from the same donor decrease the risk of GVHD. In comparison to standard Donor Lymphocyte Infusion (DLI), with a 40–60% risk of acute GVHD, early studies show minimal GVHD [130]. Recently, in a study of 20 subjects suffering from B cell cancer the patients received CD19 CAR T cells that were received from the same donor, 8 of 20 patients who received treatment obtained remission, and no GVHD was reported [132].

### 6.2. Virus-Specific CAR-T Cells

The main goal of using virus-specific T cells for manufacturing CAR is to have a non-alloreactive TCR. Viral specific T cells were developed following transplantation for viral infections therapy [127], despite there not being any HLA matching, but with minimal GVHD; therefore, they are a hopeful source for allogeneic CAR-T cell production [130].

### 6.3. Gene Editing

An “off-the-shelf” CAR-T cell derived from a healthy, allogenic donor can be used for every patient, eliminating the limitations of HLA matching [133]. These CAR-T cells have been generated by knocking out the endogenous TCR. For expressing TCRαβ both alpha and beta chains, a single gene code for the alpha chain (TRAC) was needed, while the beta chain was encoded by two genes, so disrupting TRAC is a preferred strategy for preventing TCR expression. By using nucleases such as zinc-finger nucleases (ZFNs), transcription activator-like effector nucleases (TALEN), and the clustered regularly interspaced short palindromic repeat (CRISPR)/Cas9 system, TCR could be excised. Once the TCR was deleted, the risk of GVHD could decrease [127]. The MHC class I (HLA-A2) could be removed on donor-derived CAR-T by the same methods to prevent rejection, this strategy inhibits donor CAR-T cell recognition by host TCRαβ via HLA class I. These HLA-A2 negative CAR-T cells were protected from CTL attack and survived in culture with target tumor cells for more than 50 days [134], and MHC absence can incite NK cells’ response to allogeneic T cells. To prevent NK-mediated cell killing induced by the loss of HLA, CD47 was involved as a further potential to improve the “don’t kill me” signal on T cells and enable them to survive [135].

## 7. Overcoming CAR-T Cell-Based Approaches Boundaries

Although CAR-T cell therapy is a promising strategy in cancer immunotherapy, there are some challenges to its application such as T-cell trafficking to the TME, fewer suitable tumor targets, low persistence of CAR-T cell within the tumor, and immunosuppressive TME [103]. Interaction between chemokines in tumor and chemokine receptors presented on T cells causes active trafficking, so mismatched tumor-induced chemokine/chemokine receptors are recognized as one of the main factors in limiting T-cell infiltration. CAR T cells have been changed to express the chemokine (C-C motif) receptor (CCR2b). The C-C Motif Chemokine Ligand 2 (CCL2) chemokine receptor is significantly expressed on the target tumors [136]. Furthermore, some studies showed IL-8 or chemokine (C-X-C motif) ligand 8 (CXCL8) receptor, C-X-C Motif Chemokine Receptor 1 (CXCR1) or CXCR2, altered CARs significantly enhance the invasion and persistence of T cells in cancer environment [137].

Finding a safe TAA that not only is enriched in cancers, but also with a low-level expression on normal tissues, is a critical step in prosperous CAR production [138]. IL-13Rα2 is one of the specific markers for glioblastoma (GBM) malignant cells that have a key role in disease prognosis. Brown et al. for the first time applied an engineered IL-13Rα2-directed CAR-T cell in three patients with recurrent GBM to evaluate the efficacy of this specific construct [139]. In an ongoing phase I clinical trial, its adverse effects are under evaluation (NCT02208362). HER2 is another target to treat GBM by a virus-specific modified CAR-T cell that was examined in a phase I clinical study with a moderate response in one patient (NCT01109095). Anti-HER2 CAR-T cells were administration in HER2 + breast cancer patients as well (NCT02442297; NCT03696030). In breast cancer, an ongoing phase I clinical study is evaluating the safety of mesothelin-specific CAR-T cells in mesothelin + breast cancer patients (NCT02792114). A mesothelin-directed CAR-T cell is also evaluated in other mesothelin + tumor cells including lung adenocarcinoma (NCT03054298; NCT02414269) and pancreatic ductal adenocarcinoma (NCT01897415; NCT03323944). c-Met is another marker that is mainly expressed in about half of the breast tumor cells. The RNA which transduced to a c-Met directed CAR-T cells was assessed in a phase I clinical trial (NCT03060356). Carcinoembryonic antigen (CEA) was targeted in order to CAR-T cell therapy in colorectal or other CEA + tumor cells. Katz et al. in a phase I clinical trial have determined the efficacy of anti-CEA CAR-T cells in patients with liver metastasis and improved tumor cell killing was observed (NCT01373047) [140]. Zhang et al. evaluated the efficacy of CEA-directed CAR-T cells in CEA + colorectal patients in a phase I clinical study and partial efficacies were found (NCT02349724) [141]. Claudin 18.2 is another marker which has expression in gastric epithelia [142]. An ongoing phase I clinical trial was evaluating the efficacy of anti-claudin 18.2 CAR-T cells in seven gastric and five pancreatic cancer patients (NCT03159819), and one complete and three partial responses were observed [143]. Prostate stem cell antigen (PSCA) targeted CAR-T cells have been administered in patients with pancreatic, stomach, or prostate cancer (NCT02744287). Table 3 aims to summarize current clinical trials about cancer immunotherapy by CAR-T cells in solid tumors. Most of these clinical trials are in the early phase of their pathways, and they are not in the active phase.

In solid tumors, the immunosuppressive signaling pathway secretes chemokines and inhibits the invasion of T cells to the tumor area. The immunosuppressive TME includes myeloid cells, the vascular system, and fibroblasts, which are immunosuppressive and inhibit the infiltration of tumor-reactive T-cells into solid cancers. [144,145,146]. Indeed, the immunosuppressive TME does not permit the CAR-T cells to infiltrate the CAR-T cells to the TME effectively. The administration of chemotherapy with CAR-T cell therapy has gained special attention. However, the considerable side effects and subsequent complications of chemotherapies do not bring well-tolerable therapy to the affected patients. Moreover, one of these kinds of clinical trials has failed to demonstrate objective responses in affected patients (NCT01218867). Investigations have also demonstrated that blocking ICs for reversing immunosuppressive TME is a hopeful approach to increase the beneficial effect of CAR-T treatment in cancers, particularly in solid tumors [147]. In pre-clinical trials, Darcy et al. examined self-antigen human epidermal growth factor receptor 2 (Her2) in transgenic mice and used two different Her2+ tumor targets to investigate anti-tumor efficacy and safety of CAR T cells and anti-PD-1 antibody in the combined form [148]. Clinical studies for the impact of blocking PD-1 on CAR T-cell action arises from hematological malignancies. Infusion of Pembrolizumab after CD19 CAR T-cell therapy in patients with progressive lymphoma resulted in a significant anti-tumor response [149]. A study has shown more effective outcomes in adding pembrolizumab and nivolumab to CAR-T cell treatment in patients with relapsed or refractory B-ALL that were pretreated with CD19 specific CAR-T cell [150]. However, some clinical and preclinical studies combine CAR-T cells with an ICI to diminish T cell’s exhaustion and increase their persistence [148,149,151] but more investigations are needed to have therapeutic benefits from this combination. In recent combinational strategies, genetically engineered CAR-T cells produce mAbs against immune checkpoints themselves. For instance, anti-CAIX (carbonic anhydrase IX) CAR-T cells have been engineered by using lentiviral vectors to secrete anti-PD-L1 mAbs in tumor sites [152]. Likewise, Li et al. have developed CAR-T cells that produce anti-PD-1 mAbs and they have demonstrated an enhanced anti-tumor activity in the human lung carcinoma xenograft mouse model [153]. Yang et al. have genetically engineered anti-PD-1 and anti-PD-L1 CAR-T cells which target PD-1/PD-L1 interactions. This study demonstrated increased persistence of CAR-T cells and a more efficient anti-tumor function by applying mentioned CAR-T cells in xenograft and orthotopic models of human pancreatic cancer [154]. Qin et al. have developed a negative dominant form of PD-1, dPD1z that comprising of two vectors, a vector including the extracellular and transmembrane domains of PD-1, and an anti-PD-L1 CAR vector, CARPD-L1z, and another vector containing a high-affinity anti-human PD-L1 scFV. The intracellular part of these two vectors containing the co-stimulation domains such as 4-1BB and Toll-like receptor 2 (TLR2), and also CD3ζ signaling domain. dPD1z and CARPD-L1z can effectively lyse PD-L1 positive tumor cells and decrease tumor cell growth in patient-derived xenograft models [155]. Despite relevant efficacy potential, ICI-based combination approaches also harbor predictable and unexpected issues. First of all, increased activation of autoreactive T cells leads to extensive autoimmune toxicity. Secondly, anti-PD-1 mAbs can be caught by PD-1 tumor-related macrophages via their Fc domain before they contact the T cell surface, reducing their capacity to inhibit PD-1 and suppressing the function of T cell. Thirdly, the effectiveness of the PD-1 mAbs blockade is short-lived and relies on repeated treatment. Lastly, resistance to checkpoint inhibitors can be developed [156]. As a particular scientific strategy to disrupting the PD-1/PD-L1 interaction genetic modification, knocking out the PD-1 gene in T cells has certain benefits; this was achieved by editing the PD-1 gene via CRISPR. Disrupting the PD-1 gene by CRISPR/Cas9 in the Glypican-3-directed CAR-T cell has demonstrated diminished CAR-T cell’s exhaustion and enhanced tumor cell killing in hepatocellular carcinoma [157]. The intrinsic PD-1 checkpoint inhibition in CAR-T cells show greater long-lasting impact and less toxicity in comparison to the administration of PD-1 antibodies, boosting the effectiveness of CAR-T therapy [147]. Cherkassky et al. have added a dominant-negative receptor (DNR) PD-1 to investigate the impact of cell-intrinsic PD-1 signaling inhibition. CD28 CD19-specific CAR-T cells co-transduced with PD-1 DNR showed the improved function of T cells in vitro and long-lasted T cells in vivo with higher efficacy and better control of tumor cells [158]. Table 4 presented ongoing clinical trials of PD-1 and CAR-T cells’ combination in leukemic disorders. Based on the comprehensive discussion of this narrative review, the engineered CAR-T cell therapy can bring optimal outcomes with the highest response rate and the lowest side effects in patients with malignancy

## 8. Future Immune Checkpoints Candidates: Boosting CAR-T Therapy Effectiveness

CAR-T therapy enhances the expression of LAG-3 in CAR-T cells in the tumor microenvironment. Zhang et al. used CRISPR/Cas9-mediated gene editing to produce CD19 CAR-T cells with LAG-3 knockout, with about 70% efficiency. Nevertheless, these cells were found to have no noticeable functional supremacy over non-edited CAR-T cells. It needs more study to investigate the efficacy of CAR-T cells with dual PD-1 and LAG-3 blocking [119], as in the enhanced expression of PD-1 and LAG-3 on activated CAR-T cells, TIM-3 expression is also enhanced [161]. Moreover, the application of PD-1 or TIM-3 blockade in CAR-T cell therapy led to an increase in synergistic anti-tumor function, indicating that TIM-3 blockage could be effective in combination with CAR-T therapy [119]. Novel bispecific CAR-T cell constructs targeting both CD13 and TIM3 have been administered in the AML xenograft model. Because of developed TIM3 in tumor cells, these bispecific CAR-T cells exhibited more efficacy in eradicating tumor cells [162]. A preclinical study showed a CD19-specific CAR-T cell with a disrupted PD-1 encoding gene by CRISPR/Cas9 can augment tumor killing in a subcutaneous xenograft model [163]. Some clinical trials are evaluating the therapeutic benefits of the CRISPR/Cas9 gene-edited CAR-T cells in mesothelin (MSLN)-positive solid tumors (NCT03545815 and NCT03747965). A different approach to diminish PD-1 expression in CAR-T cells and prevent unwanted mutations that maybe happened by using CRISPR/Cas9 which leads to CAR-T cells misfunction is to reduce N-linked glycosylation of PD-1. In this strategy edited CAR-T cells have shown improved cytotoxic activity in vitro and in vivo [164].

## 9. Conclusions

Over past decades, remarkable progress in cancer immunotherapies has been made, such as developing ICIs, BiTE, and CAR-T cells. Although developing mAbs against immune checkpoints axes has improved anti-tumoral immune responses, their applications have been associated with a remarkable risk for autoimmunity development. Since macrophages can cleave these mAbs, targeting immune checkpoints via mAbs cannot bring a long-lasting anti-tumoral immune response. Although BiTE can direct specific CD8+ T cells to the TME, the risk of tumor relapse remains a daunting challenge in this approach. Since CAR-T cells recognize tumor antigens, independent from the MHC complex, dysfunctional antigen-presenting cells can not impede the development of robust anti-tumoral immune responses. However, the immunosuppressive nature of TME, which is mainly induced by immune checkpoints, has posed a threat to the recruitment of CAR-T cells to TME. The genetic engineering of CAR-T cells, to remove immune checkpoint related genes, is a novel and promising approach to counteract the immunosuppressive TME in patients with cancer. It can be concluded that from applying learned lessons of the last clinical trials and different immunotherapy approaches, we can bring opportunities to reach a treatment with the highest response rate and the lowest side effects for patients with cancer.

## Figures and Tables

**Table 1:** FDA approved Immune checkpoint inhibitors for different types of cancers [32].

Cancer Type	FDA Approved Drug	FDA Approval Year
Melanoma	Ipilimumab	2011
	Nivolumab	2014
	Pembrolizumab	2014
	Nivolumab + Ipilimumab	2015
Pediatric Melanoma	Ipilimumab	2017
Adjuvant (pre-surgical) treatment for stage III Melanoma	Pembrolizumab	2019
NSCLC	Pembrolizumab	2015
	Nivolumab	2015
	Atezolizumab	2016
	Durvalumab	2018
First-line treatment of patients with stage III NSCLC	Pembrolizumab	2019
NSCLC	Atezolizumab (in combination with chemotherapy)	2019
Hodgkin lymphoma	Nivolumab	2016
Hodgkin lymphoma (adult and pediatric patients)	Pembrolizumab	2017
Urothelial carcinoma	Atezolizumab	2016
	Nivolumab	2017
	Durvalumab	2017
	Avelumab	2017
	Pembrolizumab	2017
HNSCC	Pembrolizumab	2016
	Nivolumab	2016
First-line treatment of patients with metastatic or recurrent HNSCC	Pembrolizumab	2019
Merkel cell carcinoma	Avelumab	2017
	Pembrolizumab	2018
MSI-HI solid tumors	Pembrolizumab	2017
	Nivolumab + Ipilimumab	2018
MSI-HI CRC	Nivolumab	2017
HCC	Nivolumab	2017
	Pembrolizumab	2018
Gastric and gastroesophageal carcinoma	Pembrolizumab	2017
Advanced ESCC	Pembrolizumab	2019
Renal cell carcinoma	Nivolumab	2015
	Nivolumab + Ipilimumab	2018
	Avelumab	2019
Cervical cancer	Pembrolizumab	2018
PMBCL	Pembrolizumab	2018
SCLC	Nivolumab	2018
	Pembrolizumab	2019
Extensive-stage SCLC	Atezolizumab	2019
CSCC	Cemiplimab	2018
TNBC	Atezolizumab	2019
Endometrial carcinoma	Pembrolizumab	2019
NMIBC	Pembrolizumab	2020
Advanced HCC	Nivolumab + Ipilimumab	2020
Extensive-stage SCLC	Durvalumab (in combination with chemotherapy)	2020
Metastatic NSCLC	Nivolumab + Ipilimumab	2020
Metastatic or recurrent NSCLC	Nivolumab + Ipilimumab (in combination with chemotherapy)	2020
Untreated HCC	Atezolizumab (in combination with Bevacizumab, an anti-VEGF-A)	2020
Unresectable advanced, recurrent or metastatic ESCC	Nivolumab	2020
Unresectable or metastatic TMB-H solid tumors	Pembrolizumab	2020
Recurrent or metastatic CSCC	Pembrolizumab	2020
MSI-H or dMMR CRC	Pembrolizumab	2020
BRAF V600 mutation-positive advanced melanoma	Atezolizumab (plus cobimetinib and vemurafenib)	2020

Abbreviations: PMBCL = primary mediastinal large B-cell lymphoma, SCLC = small cell lung cancer, MSI-H = microsatellite instability-high, NSCLC = non-small cell lung cancer, CSCC = cutaneous squamous cell carcinoma, HNSCC = head and neck squamous cell carcinoma, CRC = colorectal cancer, NMIBC =non-muscle invasive bladder cancer, TNBC =triple-negative breast cancer, HCC = hepatocellular carcinoma, ESCC = esophageal squamous cell carcinoma, TMB-H = tumor mutational burden-high, dMMR = mismatch repair deficient.

**Table 2:** Summary of the biological function of immune checkpoint inhibitor classes.

Molecules	Ligands	Receptor Expression	Function	Drugs	References
CTLA-4	B7-1(CD80), B7-2(CD86)	Activated T cells, Tregs	Co-inhibition	Ipilimumab *, Tremelimumab	[33,34,41]
PD-1	PD-L1,PD-L2	TILs, B cells, Effector T cells, Tregs NK cells macrophages, subsets of DC	Co-inhibition	Nivolumab *, Pembrolizumab * Cemiplimab *	[49,50,51,52]
PD-L1	PD-1, B7-1	DCs, T cells monocytes, macrophages, mast cells, B cells, NK cells	Attenuate development of T cells in inflamed tissues	Atezolizumab *, Avelumab *, Durvalumab *	[49,50,51,52]
LAG3(CD22)	MHC-II, LSECtin, Galectin-3	Activated T cells, B cells, Tregs, NK cells, DCs	Negative regulation of T-cell expansion, DC activation	IMP321/Eftilagimod alpha, Relatlimab /BMS- 986016, LAG525, MK-4280, Sym022, REGN3767, TSR-033	[53,54,55,56,57]
TIM3 (HAVCR2)	Galectin9, PtdSer, HMGB1, CEACAM-1	Activated T cells, NK cells, DCs, B cells, Tregs, monocytes	Maintaining peripheral tolerance	TSR-022, MBG453, Sym023, INCAGN2390, LY3321367, BMS-986258, SHR-1702,	[22,58,59,60]
TIGIT (WUCAM/ Vstm3/Vsig)	CD155, CD112	NK Cells, T cells	Negative regulation of T cells activity, DC tolerization	MK-7684, Etigilimab /OMP-313 M32, AB-154, Tiragolumab/ MTIG7192A/RG-6058, BMS-986207, ASP-8374	[31,60,61,62,63]
VISTA (PD-1H/ DD1α/Gi24/ Dies1/B7-H5)	VSIG-3	T Cells, Myeloid cells	T-cell negative regulation; CD4 + T cells suppression	JNJ-61610588 CA-170	[22,31,64,65,66,67]
B7-H3 (CD276)	Unknow	Activated T cells, DCs, NK cells, tumor tissue monocytes	Co-inhibition	Enoblituzumab /MGA271, MGD009, 8H9	[68,69,70,71,72]
BTLA (CD272)	HVEM	Mature B cells, Tregs, T cells, DCs, macrophages	Co-inhibition	TAB004/JS004	[31,73,74,75]

* FDA Approved.

**Table 3:** Clinical trials regarding CAR-T cells.

Interventions	Mechanism of Action	Cancer	Clinical Trial Phase	Study Start Date	The Status	ClinicalTrials.gov Identifier
Anti-GD2, PSMA, Muc1, or Mesothelin	Recognition of the tumor-related antigens via ex vivo training of T-cell with GD2, mesothelin, PSMA, and Muc-1	Cervical cancer	Phase I/II	2017	Recruiting	NCT03356795
huCART-meso cells	ex vivo training of T-cell and depletion of lymphocytes	Pancreatic cancer	Phase I	2017	Active, not recruiting	NCT03323944
Anti-TM4SF1 and EpCAM CAR-T therapy	Recognition of the tumor-related antigens via ex vivo training of T-cell with TM4SF1 and EpCAM	Advanced solid neoplasia	Not Applicable	2019	Not yet recruiting	NCT04151186
Anti-meso CAR-T cells in combination with Fludarabine and Cyclophosphamide	ex vivo training of T-cell to identify mesothelin. DNA synthesis inhibition. Protein synthesis inhibition	Ovarian cancer	Phase I	2019	Recruiting	NCT03799913
Anti-CD19, CD20, CD22, CD30, CD38, CD70, and CD123 4th generation CAR-T cells	ex vivo training of T-cell to identify CD19, CD20, CD22, CD30, CD38, CD70, and CD123	B cell malignancies	Phase I/II	2017	Recruiting	NCT03125577
Anti-VEGFR2 CAR-T cells therapy in combination with cyclophosphamide, Aldesleukin, and Fludarabine	ex vivo training of T-cell against VEGFR2, inhibiting tumor growth, upregulating T cell production, and suppression of tumor growth, respectively	Metastatic cancers, metastatic melanoma, and renal cancer	Phase I/II	2010	Terminated (no objective responses)	NCT01218867
Anti-CEA CAR-T cells	Recognition of the tumor-related antigen via ex vivo training of T-cell with the carcinoembryonic antigens	Liver metastases and pancreatic cancer	Phase I	2017	Active, not recruiting	NCT02850536
Anti-CEA CAR-T cells	Recognition of the tumor-related antigen via ex vivo training of T-cell with the carcinoembryonic antigens	- Peritoneal carcinomatosis - Peritoneal metastases - Colorectal cancer - Gastric cancer - Breast cancer - Pancreas cancer	Phase I	2018	Active, not recruiting	NCT03682744
Anti-CD147 CAR-T cell	Recognition of the tumor-related antigen via ex vivo training of T-cell with the CD147	Advanced hepatocellular carcinoma	Phase I	2019	Recruiting	NCT03993743
Anti-CD147 CAR-T cell	Recognition of the tumor-related antigen via ex vivo training of T-cell with the CD147	Recurrent glioblastoma	Early Phase I	2019	Recruiting	NCT04045847
Anti-EGFR806 CAR-T cell	Recognition of the tumor-related antigen via ex vivo training of T-cell with the EGFR806	- Central nervous system tumor - Pediatric glioma - Ependymoma - Medulloblastoma -Germ cell tumor - Atypical teratoid/rhabdoid tumor - Primitive - Neuroectodermal tumor - Choroid plexus carcinoma - Pineoblastoma	Phase I	2019	Recruiting	NCT03638167
Anti-Muc-1 CAR-T cells	Recognition of the tumor-related antigens via ex vivo training of T-cell with Muc-1	Intrahepatic cholangiocarcinoma	Phase I/II	2018	Recruiting	NCT03633773
Anti-EpCAM CAR-T cells	Recognition of the tumor-related antigens via ex vivo training of EpCAM	- Nasopharyngeal carcinoma - Breast Cancer	Phase I	2016	Recruiting	NCT02915445
Anti-mesothelin CAR-T cells	ex vivo training of T-cell to recognize mesothelin.	Pancreatic cancer	Phase I	2017	Active, not recruiting	NCT03323944
Anti-GPC3 CAR-T cell	CAR-T cells against Glypican-3 positive hepatocellular tumor cells	Hepatocellular carcinoma	Phase I	2016	Recruiting	NCT02905188

Abbreviations: CAR-T: chimeric antigen receptor T, PSMA: prostate-specific membrane antigen, Muc-1: mucin 1, TM4SF1: transmembrane 4 l six family member 1, EpCAM: epithelial cell adhesion molecule, VEGFR2: Vascular endothelial growth factor receptor 2, CEA: Carcinoembryonic antigen, EGFR: epidermal growth factor receptor, GPC3: Glypican-3.

**Table 4:** Ongoing clinical trials of PD-1 and CAR-T cells combination in hematological malignancies [159,160].

NCT Number	Type of Malignancy	Status	Location	Summary of Study
NCT03287817	-DLBCL -Relapse/Refractory DLBCL	Recruiting	USA	A phase I/II study aiming to evaluate the efficacy of AUTO3 (anti CD19, CD22 CAR-T cell) followed by anti PD-1 antibody for limited time
NCT04213469	B cell lymphoma	Recruiting	China	Evaluating the efficacy of PD-1 knockout CD19-directed CAR-T cell
NCT02650999	-DLBCL -Follicular lymphomas -Mantle cell lymphomas	Active, not recruiting	USA	Phase I/II study of pembrolizumab in patients with relapsed/refractory lymphoma after CTL019
NCT03298828	-Acute lymphoblastic leukemia -Burkitt Lymphoma	Not yet recruiting	China	A phase I study determining the efficacy of CD19 CAR and PD-1 knockout engineered T cells
NCT03932955	Relapsed/refractory B cell lymphoma	Recruiting	China	A phase I study evaluating the efficacy and safety of MC-19PD-1 CAR-T cells
NCT03208556	Relapsed/refractory B cell lymphoma	Unknown	China	A phase I study determining the efficacy and safety of iPD-1 CD19-CAR-T cells
NCT03540303	Relapsed non-Hodgkin lymphoma	Unknown	China	A phase I study assessing the efficacy and safety of Cytoplasmic activated PD-1 CAR-T cells
NCT04163302	Relapsed/refractory B cell lymphoma	Recruiting	China	A phase II study determining the efficacy and safety of CD19-PD-1 CAR-T cells
NCT04162119	Relapsed/refractory Multiple myeloma	Recruiting	China	A phase II study evaluating the safety and efficacy of BCMA-PD1-CART cells
NCT04134325	Relapsed/refractory Hodgkin lymphoma	Recruiting	USA	An early phase I study determining the efficacy of Nivolumab and Pembrolizumab after anti-CD30 CAR-T cell therapy
ChiCTR-OIC-17011310	Refractory/aggressive non-Hodgkin lymphoma	Recruiting	China	A phase I/II study determining the efficacy of dPD-1 hCD19CAR-T cells
ChiCTR1800020306	Relapsed/refractory B cell lymphoma	Recruiting	China	A phase II study assessing the efficacy and safety of PD-1 knockdown engineered anti-CD19 CAR-T cells
ChiCTR1800018713	Relapsed/refractory non-Hodgkin lymphoma	Recruiting	China	Evaluating the efficacy and safety of PD-1 knock out CD19/CD20/CD22/CD30 directed CAR-T cells

Abbreviations: CAR-T: chimeric antigen receptor T, PD-1: programmed cell death protein 1, BCMA: B-cell maturation antigen.
